# Comparison of Pulmonary and Systemic NO- and PGI_2_-Dependent Endothelial Function in Diabetic Mice

**DOI:** 10.1155/2018/4036709

**Published:** 2018-06-04

**Authors:** Andrzej Fedorowicz, Elżbieta Buczek, Łukasz Mateuszuk, Elzbieta Czarnowska, Barbara Sitek, Agnieszka Jasztal, Antonina Chmura-Skirlińska, Mobin Dib, Sebastian Steven, Andreas Daiber, Stefan Chlopicki

**Affiliations:** ^1^Jagiellonian Centre for Experimental Pharmacology (JCET), Jagiellonian University, Bobrzyńskiego 14, 30-348 Kraków, Poland; ^2^Chair of Pharmacology, Jagiellonian University Medical College, Grzegórzecka 16, 31-531 Kraków, Poland; ^3^Department of Pathology, The Children's Memorial Health Institute, Al. Dzieci Polskich 20, 04-730 Warsaw, Poland; ^4^Center for Cardiology 1, Laboratory of Molecular Cardiology, University Medical Center of the Johannes Gutenberg University, Mainz 55131, Germany

## Abstract

Diabetes increases the risk of pulmonary hypertension and is associated with alterations in pulmonary vascular function. Still, it is not clear whether alterations in the phenotype of pulmonary endothelium induced by diabetes are distinct, as compared to peripheral endothelium. In the present work, we characterized differences between diabetic complications in the lung and aorta in db/db mice with advanced diabetes. Male, 20-week-old db/db mice displayed increased HbA1c and glucose concentration compatible with advanced diabetes. Diabetic lungs had signs of mild fibrosis, and pulmonary endothelium displayed significantly ultrastructural changes. In the isolated, perfused lung from db/db mice, filtration coefficient (K_f,c_) and contractile response to TXA_2_ analogue were enhanced, while endothelial NO-dependent modulation of pulmonary response to hypoxic ventilation and cumulative production of NO_2_^−^ were impaired, with no changes in immunostaining for eNOS expression. In turn, 6-keto-PGF_1*α*_ release from the isolated lung from db/db mice was increased, as well as immunostaining of thrombomodulin (CD141). In contrast to the lung, NO-dependent, acetylcholine-induced vasodilation, ionophore-stimulated NO_2_^−^ generation, and production of 6-keto-PGF_1*α*_ were all impaired in aortic rings from db/db mice. Although eNOS immunostaining was not changed, that of CD141 was clearly lowered. Interestingly, diabetes-induced nitration of proteins in aorta was higher than that in the lungs. In summary, diabetes induced marked ultrastructural changes in pulmonary endothelium that were associated with the increased permeability of pulmonary microcirculation, impaired NO-dependent vascular function, with compensatory increase in PGI_2_ production, and increased CD141 expression. In contrast, endothelial dysfunction in the aorta was featured by impaired NO-, PGI_2_-dependent function and diminished CD141 expression.

## 1. Introduction

Diabetes induces profound alterations in systemic circulation and is the leading cause of macro- and microangiopathies such as diabetic retinopathy, nephropathy, and myocardial infarct, as well as peripheral artery disease [1–3]. The detrimental effects of diabetes in the lungs are less clinically apparent. However, epidemiological and experimental data suggested that insulin resistance and diabetes affect the lung. In diabetic subjects, the risk of pulmonary hypertension and pulmonary embolism was increased [4]. Interestingly, several studies have suggested that diabetes results in the impairment of respiratory function [5–7] and increased susceptibility to allergic response/inflammation induced with LPS or airway bacterial infection [8–10]. Structural changes in blood-alveolar barrier and diffusion impairment *in vivo* have also been reported [11, 12]. Despite these reports, the possible detrimental effects of insulin resistance and diabetes on the pulmonary circulation have received little attention, and therefore research on this aspect of the pathophysiology of diabetes has largely been neglected. To the best of our knowledge, there are only few reports directly comparing systemic and pulmonary circulation response to diabetes in the same experimental model. Such an approach could give a better understanding of similarities and differences between diabetes-induced changes in pulmonary and peripheral circulation [13, 14].

Furthermore, although peripheral endothelial dysfunction represents a well-recognized hallmark of peripheral diabetic macro- and microangiopathies [2, 7, 15], the evidence on the development of pulmonary endothelial dysfunction in diabetes is rather conflicting. Both the presence and lack of impairment of NO-dependent pulmonary endothelial function have been reported [13, 16, 17]. In turn, an increase in pulmonary microvascular permeability without changes in eNOS or with increased iNOS expression has also been demonstrated [13, 16–18].

Similarly, reports on diabetes-induced changes in PGI_2_ production in the pulmonary circulation are also not consistent. In streptozotocin-treated rats, basal PGI_2_ production and stimulated PGI_2_ production in pulmonary circulation were reported to increase or to remain unchanged [14, 19, 20]. Interestingly, PGI_2_ is a major regulator of the expression of thrombomodulin (CD141) [21, 22], which complexes with thrombin (IIa) and activates protein C to act as an anticoagulant and endothelial protective mediator [23]. Thus, the changes in the activity of PGI_2_ may result in the alteration in the activity of thrombomodulin, despite the fact that previous studies reported no changes [24–26].

Given the nonconsistent literature, the aim of the present work was to characterize changes in pulmonary endothelial function in comparison with changes in peripheral endothelial function in the aorta, with special focus on NO- and PGI_2_-dependent pathways. For this purpose, male db/db mice at the age of 20 weeks with features of advanced diabetes were used, and pulmonary and peripheral endothelial functions and NO and PGI_2_ activities were analyzed in the isolated, perfused diabetic lung, or in the aortic rings, respectively.

## 2. Material and Methods

### 2.1. Animals

20-week-old db/db (BKS.Cg-Dock7m+/+LeprdbJ) and C57BL/6J mice, purchased from Charles River Laboratories, were housed in specific pathogen-free conditions (SPF) and fed with a standard laboratory diet and water *ad libitum*.

All experimental procedures used in the present study were conducted according to the Guidelines for Animal Care and Treatment of the European Communities and the Guide for the Care and Use of Laboratory Animals published by the US National Institutes of Health (NIH Publication number 85-23, revised 1996). All procedures were approved by the local Jagiellonian University Ethical Committee on Animal Experiments (number 53/2009).

### 2.2. Blood Count, HbA1c, and Basal Biochemistry in Plasma

Blood was collected from anaesthetized animals (pentobarbital, 140 mg/kg, i.p.) via the right ventricle to a syringe with nadroparine (end concentration: 10 U/ml) for analysis of blood count and HbA1c, and the rest of the sample were centrifuged to obtain plasma (1000*g*, 5 min, 4°C). Complete blood count was analyzed within 15 minutes after collection (by automatic blood counter ABC Vet, HORIBA). HbA1c and total hemoglobin concentrations were measured using a biochemical analyser (ABX Pentra 400, HORIBA), and the ratio was given as a percentage of HbA1c. Glucose, aspartate aminotransferase, alanine aminotransferase, creatinine, albumin, and total protein were measured using colorimetric methods (ABX Pentra 400, HORIBA).

### 2.3. Histological Analysis of the Lungs

The lungs were removed under anaesthesia and fixed in 4% buffered formalin (24 h) and were then dehydrated, embedded in paraffin, cut into 5 *μ*m sections on Accu-Cut® SRM™ 200 Rotary Microtome, and stained with either hematoxylin and eosin (H&E), Masson Trichrome, Orcein and Methyl Scarlet Blue (OMSB [27]), or Picro Sirius Red. Light microscopic examination and photographic documentation were performed using an Olympus BX53F microscope equipped with a digital camera.

### 2.4. Assessment of Changes in Lung Ultrastructure

The chest of anaesthetized rats was opened, and samples of lung tissue were cut and fixed immediately using a mixture of 2.5% glutaraldehyde and 2% freshly prepared paraformaldehyde in 0.1 mol/L cacodylate buffer at pH 7.4. The lung tissue was fixed for 12 h at 4°C. Then, the lungs were postfixed in buffered 2% osmium tetroxide, dehydrated in a graded ethanol series and propylene oxide, and embedded in Epon 812. The ultrathin sections were stained according to routine protocol with uranyl acetate and lead citrate and were examined and documented by transmission electron microscopy (Jem 1011, JEOL, Japan).

### 2.5. Immunohistochemistry of Lung Tissue

After excision, lung tissues were fixed with 4% formalin solution (10 min) and placed in 50% OCT for cryopreservation (24 h), then snap frozen at −80°C. Blocks were cut into 10 *μ*m-thick cross-sectional slides. 5% normal goat serum (Jackson Immuno) or 2.5% horse serum (Vector Labs) and 2% filtered dry milk were applied to minimalize nonspecific binding of antibodies. For indirect immunohistochemical detection of von Willebrand factor (vWF), thrombomodulin (CD141), endothelial nitric oxide synthase (eNOS), vascular cell adhesion molecule 1 (VCAM-1), and macrophage content (MAC3), sections were incubated with rabbit anti-vWF polyclonal Ig (Abcam), rat polyclonal anti-CD141 Ig (BD Bioscience), mouse monoclonal anti-eNOS Ig (BD Bioscience), rat anti-VCAM-1 monoclonal Ig (Millipore), or rat anti-MAC3 monoclonal Ig (Thermo), respectively (1 h). Antibodies were applied at concentrations of 5 *μ*g/ml or 10 *μ*g/ml (dilution 1 : 100–1 : 300 of stock solution). After rinsing in PBS, secondary biotinylated horse anti-rabbit (Vector Labs), goat anti-mouse, goat anti-rat, or goat anti-rabbit (Jackson Immuno) antibodies were applied for 30 min. At a third step of staining, Cy3-conjugated streptavidin (Jackson Immuno) and Hoechst 33258 solution were used.

### 2.6. Assessment of Endothelial Function in the Isolated Lung Preparation

Trachea in anesthetized mice were cannulated, and the lungs were ventilated with positive pressures at a rate of 90 breaths/min (VCM module from Hugo Sachs Electronic (HSE)). After laparotomy, the diaphragm was cut and nadroparine at a dose of 600 I.U. was injected into the right ventricle to prevent microthrombi formation during the surgical procedure. Then, the animals were exsanguinated by incision of the left renal artery. The lungs were exposed via a median sternotomy. The pulmonary artery and left atrium were cannulated via the right and left atrium, respectively.

Immediately after cannulation, the lung/heart block was dissected from the thorax. Using tracheal cannula, the isolated lung was mounted in a water-jacketed (38°C), air-tight glass chamber (HSE) and ventilated with negative pressures. The lungs were perfused with low-glucose DMEM with 4% albumin and 0.3% HEPES; the pH of perfusate was maintained at 7.35 throughout the whole experiment by continuous addition of 5% CO_2_ to the inspiratory air, using a peristaltic pump (ISM 834, HSE) at a constant flow (CF) of about 1.50 ml/min. The venous pressure was set between 2 and 5 cmH_2_O. The end-expiratory pressure in the chamber was set to be −3 cmH_2_O, and inspiratory pressure was adjusted between −6 and −10 cmH_2_O to yield the initial tidal volume (TV) of about 0.2 ml. Breathing frequency was set to be 90 breaths/min, and a duration of inspiration versus expiration was 1 : 1 in each breath. Every 5 min throughout the experiments, a deep breath of end-inspiratory pressure of −21 cmH_2_O was automatically initiated by VCM module (HSE) to avoid atelectasis. Airflow velocity was measured with a pneumotachometer tube connected to a differential pressure transducer (HSE), from which the value of respiratory tidal volume was determined. In experiments with constant pressure perfusion (CP), CP mode was turned on just after placing the lungs in the artificial thorax. The PAP was set to be around 3 cmH_2_O. The venous pressure was set between 2 and 5 cmH_2_O.

Both arterial and venous pulmonary pressures (PAP, PVP) were continuously monitored by ISOTEC pressure transducers (HSE) connected to a perfusion line on arterial and venous sides, respectively. The weight of the lungs was monitored by a weight transducer (HSE). TC, PAP, PVP, and lung weight data were acquired by the PC transducer card and subsequently analyzed by Pulmodynpulmo software (HSE).

All lung preparations were allowed to equilibrate for the first 15 min of perfusion with fresh buffer until baseline PAP, PVP, TV, and weight were stable. At this time point, weight of the lung (the value of which varied considerably between experiments) was set to zero.

#### 2.6.1. Hypoxic Pulmonary Vasoconstriction (HPV)

HPV was evoked by 10-minute intervals of hypoxic ventilation with a mixture of 95% N_2_ and 5% CO_2_. HPV, measured as changes in PAP, was stabilized after 5 minutes. After cessation of acute hypoxia, PAP returned to a basal level. There was a 10-minute interval of normal ventilation between HPV procedures. HPV was repeated twice, then L-NAME (300 *μ*M) was added to the perfusate and recirculated through the lung for 10 minutes, and HPV response was repeated twice again. Although TV, PAP, PVP, and weight were continuously monitored throughout the experiment, for data analysis, only maximum increase in PAP (ΔPAP) elicited by HPV was taken. TV, PVP, and weight did not change significantly during HPV.

#### 2.6.2. Vasoreactivity

After equilibration, U46619 (1 *μ*M) was added to the perfusate, which resulted in an increase of PAP, but other parameters of isolated lungs did not change. For data analysis, only maximum increase of PAP (ΔPAP) was taken.

#### 2.6.3. Pulmonary Microcirculation Permeability

In equilibrated isolated lung, perfused with constant pressure, the pulmonary venous pressure was increased to obtain PVP 1.5 cmH_2_O above PAP and was maintained at this level through 15 minutes. This resulted in an increase in weight of the lungs; other parameters were stable. After 15 minutes, PVP was set to basal value, and the process was repeated. The filtration coefficient (K_f,c_) was calculated based on recordings for 5 minutes after PVP increase [28].

#### 2.6.4. Biochemical Measurements

6-keto-PGF_1*α*_ and NO_2_^−^/NO_3_^−^ concentrations were measured in samples of effluents collected after 15 minutes of equilibration of isolated lungs perfused with constant flow, and then 5 and 45 minutes after recirculation of the perfusate was started. To assess the enzymatic source of 6-keto-PGF_1*α*_, a sample was taken before and after administration of COX-2 selective inhibitor (DuP-697, 1 *μ*M) or nonselective COX-1/COX-2 inhibitor (indomethacin, 1 *μ*M).

### 2.7. Assessment of Endothelial Function in the Isolated Aortic Rings

The thoracic aorta was quickly dissected out of the chest of anaesthetized mice, and the surrounding fat/connective tissue was removed in Krebs-Henseleit (KH) solution (mM: NaCl 118.0, CaCl_2_ 2.52, MgSO_4_ 1.16, NaHCO_3_ 24.88, K_2_PO_4_ 1.18, KCl 4.7, glucose 10.0, pyruvic acid 2.0, and EDTA 0.5). Then, the aorta was cut into 2-3 mm rings, which were mounted between two pins filled with 5 ml of KH solution chambers (37°C, pH 7.4, gassed with carbogen: 95% O_2_, 5% CO_2_) of wire myograph (620 M, Danish Myo Technology, Denmark). The unstretched aortic rings were allowed to equilibrate for 30 minutes. Then, the resting tension of the rings was increased stepwise to reach 10 mN, and the rings were washed with fresh KH solution and incubated to equilibrate for the next 30 mins.

After equilibration, the viability of the tissue was examined by contractile responses to potassium chloride (KCl 30 mM, 60 mM), and then the aortic rings were contracted with phenylephrine (Phe 0.01–3.0 *μ*M) to obtain maximal possible constriction of the rings. All tissue responses were recorded, using a data acquisition system and recording software (PowerLab, LabChart, and ADInstruments, Australia). The aortic rings were next contracted with phenylephrine to obtain 80–90% of maximal contraction, and the endothelial-dependent response was assessed using cumulative concentrations of acetylcholine (ACh 0.01–10 *μ*M). After washout, the vessels were again contracted with phenylephrine, and endothelial-independent vasodilation to cumulative concentrations of sodium nitroprusside (SNP 0.001–1 *μ*M) was assessed. The relaxation response was expressed as a percentage of the precontraction induced by phenylephrine.

### 2.8. Assessment of Prostacyclin (PGI_2_) Production in the Isolated Aortic Rings

The concentration of PGI_2_, produced by aortic rings, was quantified on the basis of the formation of 6-keto-PGF_1*α*_, a stable metabolite of PGI_2_. The aorta rings were preincubated for 15 minutes on the thermoblock (Liebisch Labortechnik) at a temperature of 37°C, in 250 *μ*l KH buffer, gassed with carbogen in the absence or in the presence of COX-2 selective inhibitor (DuP-697, 1 *μ*M) or nonselective COX-1/COX-2 inhibitor (indomethacin, 5 *μ*M). All inhibitors were dissolved in DMSO, and then control rings were incubated with addition of the same amount of DMSO (1 *μ*l/ml).

Aortic rings were then incubated for 60 minutes, and samples of effluents were collected after 3 and 60 minutes. After the experiment, aortic rings were dried (1 h, 50°C) and weighed. 6-keto-PGF_1*α*_ concentration in the effluents was measured using an EIA kit (Enzo, Life Technologies). Results were expressed as the change in 6-keto-PGF_1*α*_ concentration between 60 and 3 minutes of ring incubation and normalized to dry weight of aortic rings (pg/ml/mg).

### 2.9. Assessment of Nitrite Production in the Isolated Aortic Rings

Basal NO production by the aorta was estimated by measurements of nitrite, a primary stable product of nitric oxide oxidation, and thus considered relevant for estimation of NO synthesis by the aortic endothelium. Segments from the aortic arch were longitudinally opened, placed in 96-well plates facing up with endothelium, and incubated for one hour in 120 *μ*l KH buffer at 37°C, using a specially designed closed chamber (BIO-V(Noxygen)) that was equilibrated with carbogen gas mixture (95% O_2_, 5% CO_2_). The nitrite concentration after back reduction to NO was measured using the gas-phase chemiluminescent reaction between NO and ozone using a Sievers^∗^ Nitric Oxide Analyzer NOA 280i. The reduction of nitrites was performed in a closed glass chamber containing a reducing agent (1% wt/vol of KI in acetic acid) to convert nitrite to NO. The independent calibration on fresh NaNO_2_ standard solution was prepared for every experiment before measurements of series of samples after each refilling of glass reaction chamber, according to the manufacturer's instructions (Sievers^∗^ Nitric Oxide Analyzer NOA 280i). The limit of detection was around 10 nM of nitrite. Multiple blank samples (without aortic rings) were used to monitor nitrite contamination in the buffer and/or by laboratory atmosphere in every set of experiments. The averaged blank signal from a blank sample in a given experiment was subtracted as a background signal. Samples were kept on ice and measured directly after experiments. Nitrite concentration was expressed as ng/ml/mg of dry weight of aortic rings.

### 2.10. Immunohistochemistry in Aorta

Dissected thoracic aorta, cleared of the surrounding fat/connective tissue in Krebs-Henseleit (KH) solution, was fixed with 4% formalin solution (10 min), embedded in 50% OCT for cryopreservation (24 h), snap frozen at −80°C, and cut in 10 *μ*m-thick cross-sectional slides for immunohistochemistry. 2.5% horse serum (Vector Labs) and 2% dry milk were applied to minimalize nonspecific binding of antibodies. For von Willebrand factor (vWF) staining, rabbit anti-mouse vWF polyclonal Ig (Abcam) was used, followed by biotinylated horse anti-rabbit Ig (Vector Labs) and Cy3-streptavidin (Jackson Immuno), as described above. Nuclei were counterstained with Hoechst 33258 (Sigma-Aldrich). Images were acquired using an Axio Observer D2 fluorescent microscope, and fluorescence parameters were analyzed automatically by Columbus software (PerkinElmer).

### 2.11. Dot Blot Analysis in Aorta and Lungs

Protein expression and modification were assessed by standard dot blot analysis using established protocols [29]. 3-Nitrotyrosine- (3NT-) positive proteins were assessed by dot blot analysis of protein homogenates in aorta and lungs, which were transferred to a Protran BA85 (0.45 *μ*m) nitrocellulose membrane (Schleicher & Schuell, Dassel, Germany) by a Minifold I vacuum dot-blot system (Schleicher & Schuell, Dassel, Germany) [30]. A mouse monoclonal 3NT antibody (1 : 1000, Upstate Biotechnology, MA, USA) was used for dot blot analysis. Detection and quantification of all blots were performed by ECL with peroxidase anti-mouse (1 : 10,000, Vector Lab., Burlingame, CA). Densitometric quantification of antibody-specific bands was performed with a ChemiLux Imager (CsX-1400 M, Intas, Göttingen, Germany) and Gel-Pro Analyzer software (Media Cybernetics, Bethesda, MD).

## 3. Statistical Analysis

Results are presented as the mean ± SEM. The normality of the results was analysed using the D'Agostino & Pearson omnibus normality test and the Shapiro-Wilk test. To calculate statistical significance, a paired Student's *t*-test, Mann-Whitney test, or unpaired Student *t*-test was used. Post hoc analysis was calculated using Dunn's multiple comparisons test.

## 4. Results

### 4.1. Basal Characteristics of db/db Mice

20-week-old db/db mice were obese (body weight: 53.77 ± 0.18 *versus* 29.5 ± 0.12 g, db/db and control, resp.; *P* < 0.05) and had increased HbA1c (15.38 ± 1.7 *versus* 4.12 ± 1.36%, db/db and control, resp.; *P* < 0.05) and fasting glucose concentration in plasma (40.64 ± 5.41 *versus* 8.66 ± 1.24 mmol/l, db/db and control, resp.; *P* < 0.05) as compared to control mice. In addition to hyperglycaemic profile, db/db mice displayed signs of liver injury (increased plasma AST, ALT, e.g., for ALT: 141.90 ± 19.68 *versus* 38.02 ± 2.80, db/db and control, resp.; *P* < 0.001) and kidney injury (increased plasma creatinine 62.90 ± 63.61 *versus* 46.74 ± 7.02 *μ*mol/(L^∗^cm^2^), db/db and control, resp.; *P* < 0.05).

### 4.2. Histology and Ultrastructure of Lungs in db/db Mice

**Figure 1:**
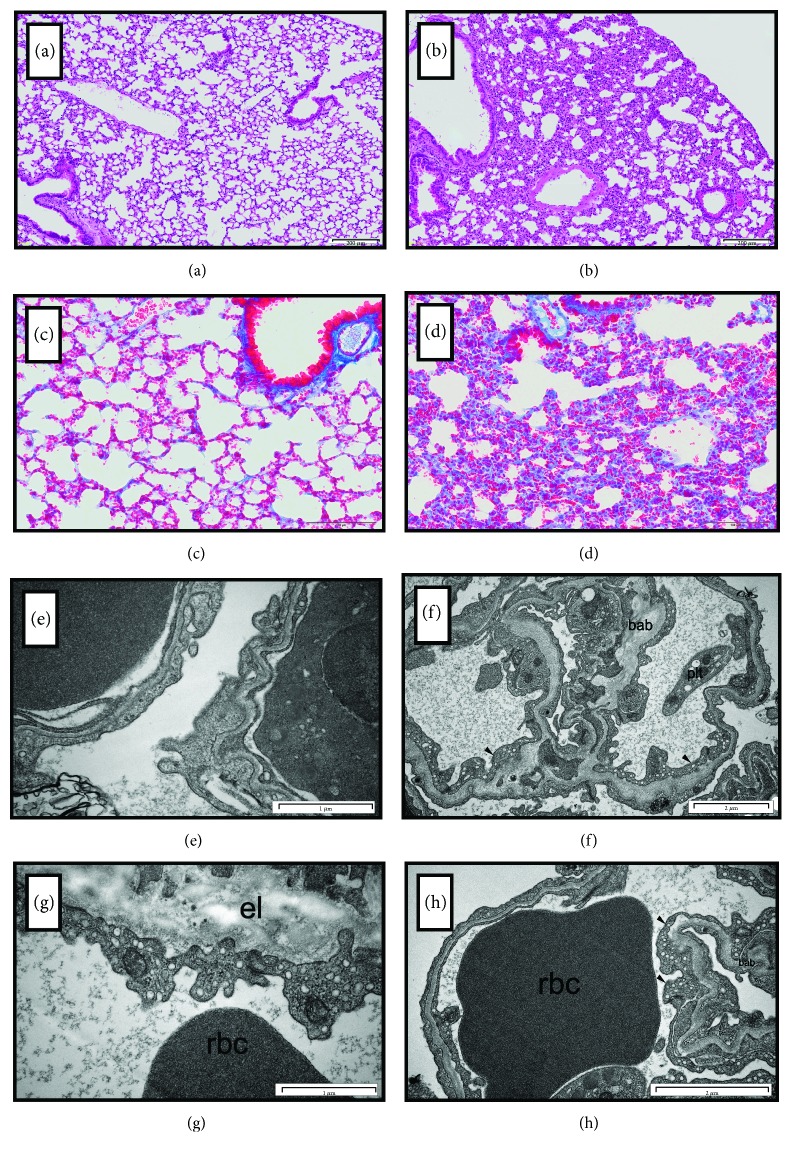
Histology of the lungs and ultrastructure of pulmonary endothelial cells from the control (a, c, e, g) and diabetic (b, d, f, h) lungs (db/db mice). (a) Histological structure of the control lungs. (b) Inflammation in the lung tissue: increased amount of cells (including granulocytes and macrophages) and (d) collagen in parenchyma of the diabetic lung tissue as compared to control; visible decreased aerial space in diabetic lungs as compared to the control (c). (e) Microphotographs of ultrastructure of the control lungs—blood-air barrier (alveolar–capillary barrier) with normal endothelial layer. (f, g, h) Microphotographs of ultrastructure of the lungs from the db/db mice. (f) Capillary endothelial cells (arrows) with numerous plasmalemmal vesicles (caveolae) on thickened blood-air barrier (bab). In the center: collapsed pulmonary alveoulus, on the right: blood platelet (plt) inside the vessel. (g) A presence of convoluted apical region in endothelial cells with cytoplasmic extensions on hyperplastic basal laminae enriched with elastine (el); on the bottom: red blood cell (rbc). (h) Endothelial cells of various heights (arrows) separated by thickened blood-air barrier (bab) from pulmonary alveolus; neighbouring red blood cell (rbc). Representative images of at least 3 independent experiments.

Lungs from db/db mice displayed inflammation, as evidenced by multicellular (including granulocytes, macrophages) infiltrations in the interstitial space (HE staining, Figure 1(b)) as compared to the control group (Figure 1(a)); mild fibrosis (increased amount of collagen), as evidenced by Trichrome staining (Figure 1(d)) as compared to the control group (Figure 1(c)); and endothelial inflammation, as evidenced by increased VCAM-1 expression in pulmonary endothelium (480,937 ± 70,112 *versus* 247,193 ± 64,821 AU, db/db and control, resp.; *P* = 0.07). Interestingly, ultrastructural investigations confirmed the presence of numerous macrophages (often lying next to each other) in the lungs from db/db mice, as compared to control mice, and they were also detected as adhering to endothelium (data not shown). Moreover, semithin sections of lung tissue revealed diminished area of alveoli (34.13 ± 4.25 *versus* 50.75 ± 3.83, db/db and control, resp.). Capillary endothelial cells displayed protruded apical regions into the capillary lumen, increased area of sarcoplasmic reticulum, plasmalemmal vesicles (caveolae), and sometimes presence of multivesicular bodies or lysosomes.

One of the typical features of db/db pulmonary microcirculation was the hyperplasia of basal lamina in db/db (Figures 1(f) and 1(h)) that was not evident in control samples (Figures 1(e) and 1(g)). Thickness of capillaries' basal lamina ranges from 0.1 *μ*m to 0.35 *μ*m, compared to 0.05 *μ*m in controls. Additionally, septa separating lung alveoli in db/db were thicker, with abundant collagen fibrils and probably contained also elastin fibrils marked by an uncontrasted area in the thin sections routinely stained with uranyl acetate and lead citrate (Figures 1(f) and 1(h)), which was also not seen in control samples (Figures 1(e) and 1(g)).

### 4.3. Alterations in Pulmonary Vascular Function and Inflammation in the Isolated, Perfused Lung from *db/db Mice*

#### 4.3.1. Changes in Basal Pulmonary Parameters and Vasoreactivity

**Figure 2:**
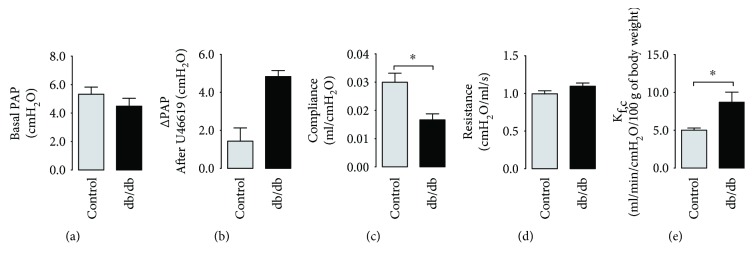
Comparison of basal parameters of the isolated, perfused lungs amongst diabetic and control animals. (a) No change in basal pulmonary pressure, although (b) enhanced reactivity to thromboxane analogue in the lungs of the diabetic mice (U-44619, 1 *μ*M, control *n* = 5, diabetes *n* = 5). (c) Decreased compliance without changes in (d) resistance of the lungs (control *n* = 5, diabetes *n* = 6). (e) Increased filtration coefficient in the diabetic pulmonary circulation (control *n* = 5, diabetes *n* = 5). Data are presented as the means ± SEM. ^∗^*P* < 0.05.

The basal pulmonary artery pressures in the isolated, perfused lungs (bPAP) were comparable in db/db and control mice (PAP: 4.70 ± 0.62 *versus* 5.20 ± 0.30 cmH_2_O, db/db and control, resp., Figure 2(a)). Vasoreactivity to thromboxane A_2_ analogue U46619 was increased threefold in the isolated lungs from db/db mice (*Δ*PAP: 4.83 ± 0.32 *versus* 1.43 ± 0.69 cmH_2_O, db/db and control, resp.; *P* = 0.073) (Figure 2(b)). Compliance but not resistance was decreased in db/db mice (compliance: 0.016 ± 0.002 *versus* 0.030 ± 0.003 ml/cmH_2_O, db/db and control, resp.; *P* < 0.05; resistance: 1.09 ± 0.04 *versus* 0.99 ± 0.04 cmH_2_O/ml/s, db/db and control, resp.; Figures 2(c) and 2(d)).

#### 4.3.2. Changes in Permeability Coefficient (K_f,c_)

In the isolated, perfused lung, an increase in pulmonary venous pressure resulted in slow, reversible weight gain of the lungs, both in control and db/db mice. Calculation of K_f,c_ revealed a higher filtration coefficient in the diabetic lungs as compared with the control lungs (8.70 ± 1.33 *versus* 5.02 ± 0.27 ml/min/cmH_2_O/100 g of body weight, db/db and control, resp.; *P* < 0.05, Figure 2(e)).

#### 4.3.3. Impairment of NO-Dependent Regulation of Hypoxic Pulmonary Vasoconstriction (HPV)

**Figure 3:**
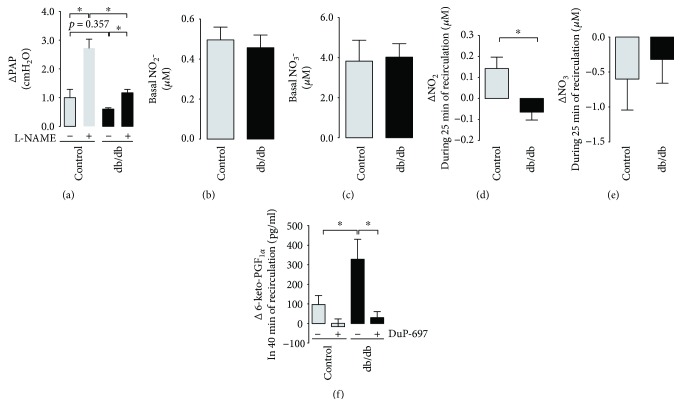
NO- and PGI_2_-dependent function in the diabetic isolated lungs. (a) Impaired NO-dependent hypoxic pulmonary vasoconstriction response after L-NAME in the lungs of the diabetic mice (control *n* = 5, diabetes *n* = 5). (b, c) Lack of change in basal production of nitrite and nitrate and (d, e) impaired capacity to cumulative production of nitrite but not nitrate in effluents from the isolated, perfused lungs of the diabetic mice (control *n* = 6, diabetes *n* = 7). (f) Increased COX-2-dependent prostacyclin production in effluents from the isolated, perfused lungs and effects of a COX-2 inhibitor (DuP-697, 1 *μ*M, control *n* = 6, diabetes *n* = 7). Data are presented as the means ± SEM. ^∗^*P* < 0.05.

In the isolated, perfused lung from control mice, episodes of hypoxic ventilation resulted in an increase of pulmonary arterial pressure (PAP) without significant changes in other parameters of isolated lung preparation (Figure 3(a)). In control lungs, the nonselective inhibitor of nitric oxide synthases, L-NAME, augmented HPV response (ΔPAP: 0.87 ± 0.32 *versus* 2.73 ± 0.44 cmH_2_O, before and after L-NAME, resp.; *P* < 0.05). However, in the isolated, perfused lung from db/db mice, the effect of L-NAME on HPV was substantially lost (ΔPAP: 0.60 ± 0.04 *versus* 1.17 ± 0.12 cmH_2_O, before and after L-NAME, resp.; *P* < 0.05) suggesting impaired NO-dependent function. L-NAME did not modify basal PAP (Δ basal PAP after L-NAME: 0.17 ± 0.03 *versus* 0.19 ± 0.06 cmH_2_O, in control and db/db mice, resp.).

#### 4.3.4. Nitrite/Nitrate (NO_2_^−^/NO_3_^−^) and Prostacyclin (PGI_2_) Production

In effluents from the isolated, perfused lungs, basal NO_2_^−^/NO_3_^−^ concentrations were comparable in both groups (e.g., NO_2_^−^ 0.40 ± 0.04 *versus* 0.56 ± 0.19 *μ*M, db/db and control, resp., Figures 3(b) and 3(c)). The cumulative concentrations of NO_2_^−^ from the diabetic lungs (see Methods for details) were significantly lower (ΔNO_2_^−^: -0.04 ± 0.04 *versus* 0.11 ± 0.05 *μ*M, db/db and control, resp.), but there were no changes in NO_3_^−^ concentrations (Figures 3(d) and 3(e)). The cumulative concentration of stable PGI_2_ metabolite, 6-keto-PGF_1*α*_, in the effluents from the isolated diabetic lungs was higher than that in the control (Δ6-keto-PGF_1*α*_: 223.5 ± 57.91 versus 95.06 ± 24.00 pg/ml, db/db and control, resp.) and was blunted after COX-2 inhibitor, DuP-697 (Figure 3(f)).

#### 4.3.5. Markers of Vascular Inflammation

**Figure 4:**
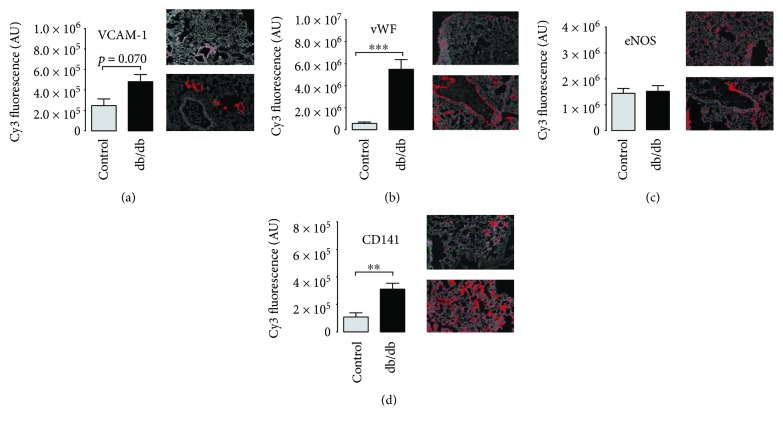
Immunohistochemical profile of the diabetic lungs. (a) Inflammation in vascular wall related to increased immunostaining of vascular cell adhesion molecule 1 (VCAM-1) and (b) von Willebrand factor in the lungs of the diabetic mice (both for VCAM-1 and vWF: control *n* = 4, diabetes *n* = 4). (c) No change in eNOS immunostaining in the diabetic lungs as compared to the control (control *n* = 4, diabetes *n* = 4). (d) Increased thrombomodulin (CD141) immunostaining in the diabetic lungs as compared to the control (control *n* = 4, diabetes *n* = 4). Data are presented as the means ± SEM. ^∗∗^*P* < 0.01, ^∗∗∗^*P* < 0.001.

In diabetic lungs, immunohistochemical staining intensity of vascular adhesion molecule-1 (VCAM-1) was increased by a trend as compared to the control samples (Figure 4(a)). Von Willebrand factor (vWF) and thrombomodulin (CD141) (but not eNOS) were higher in the diabetic lungs than in the control lungs (Figures 4(b)–4(d)). All together these results support an increased inflammatory state in the pulmonary system of diabetic mice.

### 4.4. Impairment of Endothelial Function and Inflammatory Markers in the Aorta of db/db Mice

**Figure 5:**
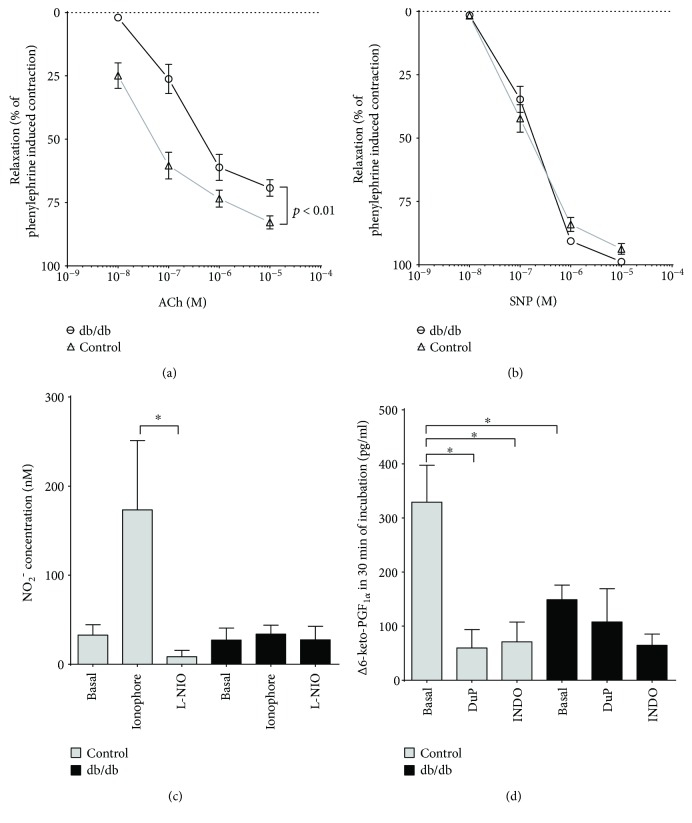
Endothelial dysfunction in systemic conduit vessel (aorta). (a, b) Impaired endothelium-dependent response to acetylcholine (ACh) with preserved endothelium-independent vasodilation in response to sodium nitroprusside (SNP) (control *n* = 6, diabetes *n* = 7). (c) Preserved basal but impaired ionophore-stimulated production of nitrite in the aortic rings of diabetic mice (control *n* = 6, diabetes *n* = 7). (d) Impaired basal production of prostacyclin as assessed by concentrations of its stable 6-keto-PGF_1*α*_ product (control *n* = 6, diabetes *n* = 6). Data are presented as the means ± SEM. ^∗^*P* < 0.05.

**Figure 6:**
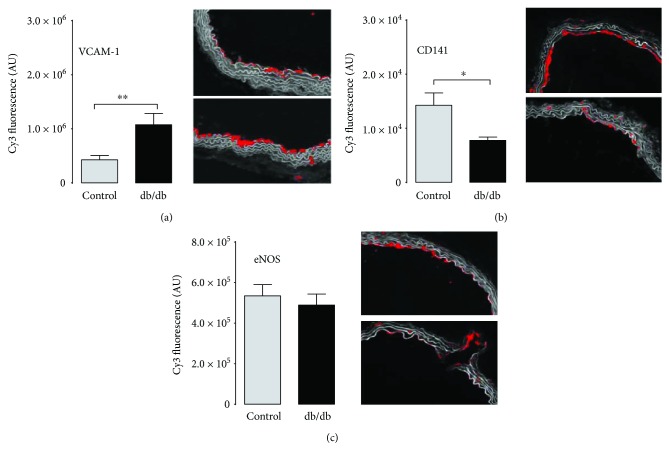
Immunohistochemical profile of systemic conduit vessel (aorta). (a) Increased VCAM-1 immunostaining intensity suggesting aortic wall inflammation in the diabetic mice (control *n* = 3, diabetes *n* = 4). (c) Comparable eNOS immunostaining intensity in the diabetic and control aortic rings (control *n* = 3, diabetes *n* = 4). (b) Decreased immunostaining intensity of thrombomodulin (CD141) in the aortic wall from the diabetic mice (control *n* = 3, diabetes *n* = 4). Data are presented as the means ± SEM. ^∗^*P* < 0.05, ^∗∗^*P* < 0.01.

Vasoreactivity to phenylephrine (30 *μ*M) in the aortic rings from db/db mice was increased (not shown). Acetylcholine- (ACh-) induced endothelium-dependent vasodilation was decreased for all concentrations in db/db mice, while sodium nitroprusside- (SNP-) induced response was preserved as compared to the control group (Figures 5(a) and 5(b)). Impairment of functional response was supported by a decline in ionophore-stimulated NO_2_^−^ production in the aortic rings from the db/db mice (33.88 ± 10.01 *versus* 173.30 ± 77.79 nM, db/db and control, resp.; *P* < 0.05). Basal NO_2_^−^ concentrations in buffer from the incubated aortic rings were comparable in both groups (27.17 ± 13.43 *versus* 32.75 ± 11.68 nM, db/db and control, resp.), whereas the effect of the NOS inhibitor, L-NIO, was striking in the aorta of the control mice and absent in the diabetic group (Figure 5(c)). COX-2-dependent production of PGI_2_ (measured as 6-keto-PGF_1*α*_ concentration in effluent) was decreased in the aortic rings from the db/db mice as compared to the control (148.80 ± 27.20 *versus* 329.30 ± 68.18 pg/ml, db/db and control, resp.; *P* < 0.05), and COX inhibitors decreased PGI_2_ production in the aorta of the control but not the diabetic mice (Figure 5(d)). Furthermore, endothelium in the aorta displayed increased VCAM-1 expression compatible with endothelial dysfunction (Figure 6(a)), although there were no changes in the eNOS immunostaining intensity (Figure 6). In turn, in contrast to the pulmonary endothelium, thrombomodulin (CD141) immunostaining intensity was decreased (Figure 6(b)).

### 4.5. Nonenzymatic Nitration in the Lungs and Aorta

**Figure 7:**
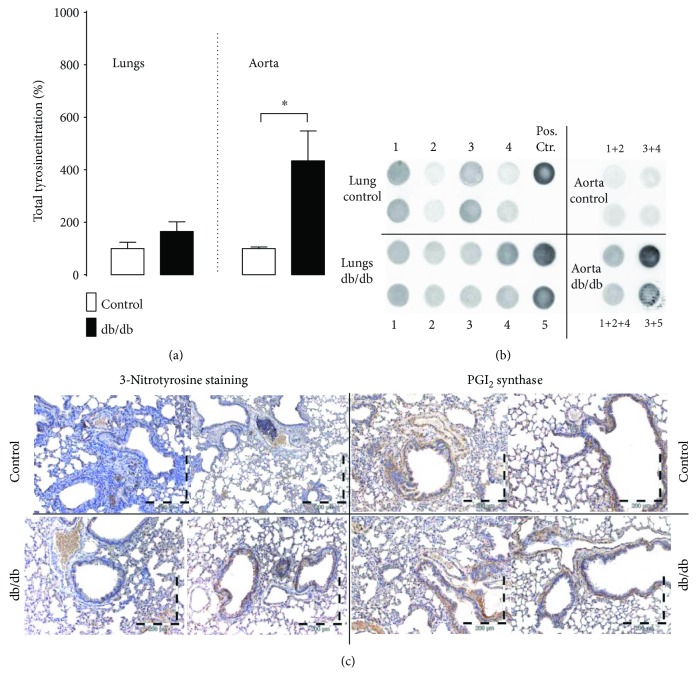
Protein nitration in the lungs and aorta. (a, b) Increased general protein nitration in the aorta but not in the lungs of diabetic mice as revealed by dot blot analysis (control *n* = 5, diabetes *n* = 5). Representative original blot images are also shown. (c) Immunohistochemical determination of 3-nitrotyrosine (3-NT) and PGIS with the slight increase in 3-NT immunostaining in the diabetic lungs—the comparison of similar regions of tissues. Data are presented as the means ± SEM. ^∗^*P* < 0.05. Representative images of at least 2 independent experiments.

Dot-blot-assessed general protein nitration was increased in the aorta but not in the lungs from the db/db mice, as compared with the control; in the lung, only a trend of increased nitration was observed (Figure 7(a) and 7(b)). Immunohistochemical staining showed only a slight increase in the signal of nitrated proteins in the lungs. A slight increase in PGIS immunostaining in diabetic lungs was also found (Figure 7(c)).

## 5. Discussion

In the present work, we characterized the phenotype of endothelial dysfunction in pulmonary endothelium, as compared with peripheral endothelium in the diabetic mice (db/db mice). We demonstrated that diabetes induced marked ultrastructural changes in pulmonary endothelium that were associated with the increased permeability of pulmonary microcirculation and impaired NO-dependent function, as well as compensatory increase in PGI_2_ production with increased thrombomodulin expression. In contrast, endothelial dysfunction in the aorta was featured by impaired NO- and PGI_2_-dependent function and diminished thrombomodulin (CD141) expression. These results suggest a differential response of pulmonary vasculature to diabetic insult in terms of PGI_2_-dependent function that might be associated with a lesser nonenzymatic protein nitration in the lung, as compared with peripheral endothelium and preserved PGI_2_ synthase activity.

Endothelial dysfunction induced by diabetes in peripheral circulation in db/db mice has been well documented [31–33] and involves (1) increased reactive oxygen species production, scavenging of endothelial NO, and increased nonenzymatic protein nitration [31, 32]; (2) decreased production of PGI_2_ and CD141 expression and impaired endothelial-dependent functional responses [31, 34–36]. Our results are in line with the previous studies as regards phenotype of endothelial dysfunction in the aorta. Importantly, we evaluated the peripheral endothelial phenotype for comparison with the analysis of the phenotype of endothelial dysfunction in pulmonary circulation that has been significantly less studied, including only few reports in the db/db mice [13, 17, 18] and studies in models of diabetes in rats [16, 37].

In the present work, we demonstrated that diabetic lungs from db/db mice displayed mild inflammatory cell infiltration and ultrastructural alterations featured by profound thickening of the basal membrane, compatible with the previous reports on diabetic lungs in humans [11, 38, 39]. Indeed, thickening of the basal membrane and an impairment of permeability of the alveolar basement membrane coexist in diabetes type II in the human lungs, and these changes are followed by a decrease in respiratory function [6, 40]. Ultrastructural changes of pulmonary endothelium reported here were also featured by activated endothelial cells that were, however, less pronounced as high-convoluted apical plasmalemma and numerous plasmalemmal vesicles reported in transgenic mice model of diabetes type I [41], suggesting milder pulmonary endothelial activation in the db/db mice. Nevertheless, we found a significant increase in endothelial permeability of diabetic pulmonary circulation, as evidenced by increase K_f,c_ measurements [28] that seem also compatible with increased permeability of the human lungs from diabetic patients [42].

The important finding of this work was the demonstration of the impairment of NO-dependent function in the lungs from the db/db mice. We took advantage of the dominant role of endogenous NO in blunting HPV [43] to study functional NO-dependent response in the whole isolated lung, instead of choosing isolated pulmonary arteries that may reflect NO-dependent function only in the selected part of the pulmonary circulation. Our original approach to detect impaired NO-dependent function was based on diminished modulatory effects of NOS inhibition on HPV response in the isolated, perfused lungs [43, 44] supported also by lowered cumulative concentrations of NO_2_^−^ in effluents from diabetic lungs. On the other hand, eNOS expression in the lungs from the control and db/db mice was not different, suggesting that alteration of the NO bioavailability was responsible for functionally impaired NO-dependent response in pulmonary circulation from the db/db mice.

Endothelium-derived PGI_2_ is often released in a coupled manner with NO [45, 46]. NO deficiency is sometimes linked with a decrease in PGI_2_ production, but in many vascular pathologies, PGI_2_ production may increase in response to nitric oxide deficiency [44, 47]. Here, PGI_2_ production in the aorta was reduced, but in the isolated lungs from the db/db mice, PGI_2_ production was augmented. The major enzymatic source of PGI_2_ in the aorta and lung was COX-2, as evidenced by the pronounced effect of COX-2 inhibition, and this is in line with the notion of COX-2 as the major source of systemic PGI_2_ [48] and important contributor to pulmonary endothelial dysfunction [49, 50]. PGI_2_ amplifies CD141 expression [21–23]. As shown here, CD141 immunointensity in the lungs was increased, while it was diminished in the aorta, which supports the link between PGI_2_ production and CD141. PGI_2_ via CD141 activates protein C, thus enhancing the anticoagulant mechanism of the vascular wall. Reciprocally, activated protein C boosts PGI_2_ production in endothelial cells [51].

Thus, pulmonary PGI_2_ affords potent antiplatelet and vasoprotective activity, activating also CD141-dependent anticoagulant mechanisms which might constitute an important compensatory mechanism in diabetes offsetting inflammatory and thrombotic processes in diabetes involving also detrimental COX-2-derived metabolites contributing to endothelial dysfunction in diabetes [49, 50, 52] .

Interestingly, 1-MNA exerts antithrombotic [53] and anti-inflammatory [54] properties mediated by the activation of COX-2 and PGI_2_ pathways. It could well be that the therapeutic efficacy of 1-MNA reported previously [53–62] is linked with the capacity of 1-MNA to stimulate compensatory mechanisms linked to pulmonary PGI_2_ [44]. Obviously, this hypothesis needs to be verified in further studies.

It is well known that diabetes is associated with increased local ROS production in intrapulmonary arteries, as well as in systemic circulation [16, 63, 64]. Superoxide anions and NO by forming peroxynitrite may lead to nonenzymatic nitration of proteins [63, 65] including PGIS; the nitration-mediated inactivation of which plays an important role in the development of endothelial dysfunction [66–69]. In systemic circulation in diabetes patients, protein nitration affects a number of enzymes, including PGIS [64, 70]. Here, we present significantly increased general nitration of protein in the aorta, and a milder effect (nonstatistically significant) was noticed in the lungs. Nonenzymatic nitration may have less significance in the diabetic lungs as compared to systemic circulation. Therefore, despite locally increased ROS generation in pulmonary vessels [16, 37, 71], in the whole lungs, ROS may not play such an important role in pulmonary circulation of the db/db mice as compared to systemic endothelium.

## 6. Conclusions

In conclusion, our results demonstrate that diabetes induced profound changes in the lung in the db/db mice involving endothelial ultrastructural changes, increased endothelial permeability, and increased vasoreactivity, as well as lung inflammation and fibrosis. Impaired NO-dependent pulmonary vascular function was associated with upregulated PGI_2_ and CD141 that might constitute an important compensatory mechanism in pulmonary circulation in diabetes that does not operate in endothelium in the aorta, whereby endothelial dysfunction is featured by impaired NO, PGI_2_, and CD141.

## Data Availability

The datasets generated during and/or analysed during the current study are available from the corresponding author on reasonable request.

## Abbreviations

6-keto-PGF_1*α*_: Stable prostacyclin metabolite
Ang: Angiotensin
AST/ALT: Aspartate transaminase/alanine transaminase
CD141: Thrombomodulin
CF: Constant flow
CP: Constant pressure
COX: Cyclooxygenase
DuP-697: Selective COX-2 inhibitor
eNOS: Endothelial nitric oxide synthase
H&E: Hematoxylin and eosin
HPV: Hypoxic pulmonary vasoconstriction
iNOS: Inducible nitric oxide synthase
1-MNA: 1-Methylnicotinamide
NO: Nitric oxide
NO_2_^−^/NO_3_^−^: Nitrite/nitrate
OMSB: Orcein and methyl scarlet blue staining
PAP: Pulmonary arterial pressure
ΔPAP: The change in pulmonary arterial pressure
PGI_2_: Prostacyclin
PVP: Pulmonary venous pressure
RVW/BW: Right ventricular to body weight ratio
SNP: Sodium nitroprusside
TV: Tidal volume
U46619: Thromboxane A_2_ analogue
vWF: von Willebrand factor.
